# Rosavin, but not its combination with salidroside, lowers total β-catenin in human osteoblast cultures: an exploratory in vitro study under mineralizing conditions

**DOI:** 10.1007/s00296-026-06278-y

**Published:** 2026-08-17

**Authors:** Piotr Wojdasiewicz, Edyta Wróbel, Maria Maślińska, Paweł Turczyn, Elżbieta U. Stolarczyk, Krzysztof Stolarczyk, Agnieszka Mikulska, Dariusz Szukiewicz

**Affiliations:** 1 Department of Biophysics, Physiology and Pathophysiology, Faculty of Health Sciences, Medical University of Warsaw, 5 Chałubińskiego Street, Warsaw, 02-004 Poland; 2 Department of Rheumatology, Rheumatology and Rehabilitation, Eleonora Reicher National Institute of Geriatrics, 1 Spartańska Street, Warsaw, 02-637 Poland; 3 Department of Early Arthritis, Rheumatology and Rehabilitation, Eleonora Reicher National Institute of Geriatrics, 1 Spartańska Street, Warsaw, 02-637 Poland; 4 Spectrometric Methods Department, National Medicines Institute, 30/34 Chełmska Street, Warsaw, 00-725 Poland; 5 Faculty of Chemistry, University of Warsaw, 1 Pasteura Street, Warsaw, 02-093 Poland

**Keywords:** Osteoblasts, Beta catenin, Wnt signaling pathway, Bone remodeling, Rhodiola

## Abstract

Osteoblast dysfunction and altered Wnt/β-catenin-dependent responses accompany bone-remodeling imbalance in rheumatic and degenerative joint disease. Rosavin and salidroside are constituents of *Rhodiola rosea* preparations used without prescription, and are always encountered together rather than separately. This exploratory in vitro study asked whether the two compounds and their combination differentially affect total β-catenin concentration and cell-covered area in human osteoblasts (HOBs). HOBs from one commercial preparation (passage 7) were cultured in growth medium (GM) or mineralization medium (MM) and exposed to rosavin 50 µM (R50), salidroside 50 µM (Sal50) or both (R50/Sal50). Total β-catenin was quantified by ELISA in four independently cultured, treated, harvested and lysed wells per condition at days 7, 14 and 21. Cell-covered area was estimated from 79 phase-contrast fields. Welch ANOVA with Games–Howell post-hoc testing was pre-specified. Total β-catenin differed between conditions at day 14 (Welch ANOVA *p* = 0.003, Holm-adjusted *p* = 0.009; η² = 0.43): 8.9 ± 3.8 ng/mL in R50 versus 28.5 ± 3.8 in Sal50, 26.5 ± 16.9 in R50/Sal50, 23.5 ± 7.9 in MM and 27.4 ± 8.6 in GM. Sal50 versus R50 was the only contrast surviving correction (difference 19.6 ng/mL, 95% CI 13.1–26.1; *p* = 0.002). No difference was detected at day 7 (*p* = 0.87) or day 21 (*p* = 0.23). Cell-covered area differed at days 7 and 21, with R50/Sal50 lowest and most variable (54.5 ± 23.4% versus 98.1 ± 1.8% in GM at day 7). Cell-covered area explained 12% of the variance in group-mean β-catenin (*r* = − 0.35). Rosavin alone was associated with a marked reduction in total β-catenin at day 14; its combination with salidroside was not. The two constituents therefore do not act interchangeably on human osteoblasts. These observations derive from a single experimental series and require replication before mechanistic or clinical interpretation.

**Supplementary Information:**

The online version contains supplementary material available at https://doi.org/10.1007/s00296-026-06278-y.

## Introduction

Pathological bone remodeling is a central component of many rheumatic and degenerative joint diseases. Inflammatory joint diseases and osteoarthritis are associated not only with cartilage damage, synovial inflammation and bone loss, but also with abnormal new bone formation, including osteophytes, enthesophytes, syndesmophytes and ectopic ossification. These processes indicate that osteoblasts should not be viewed solely as matrix-producing cells, but rather as active participants in the osteoimmune and remodeling network of the joint [1–10].

Among the pathways governing osteoblast function, the canonical Wnt/β-catenin pathway is central, contributing to differentiation, survival, matrix-related activity and skeletal homeostasis [11–18]. Its role in joint disease is context-dependent: insufficient osteoblast-mediated repair may sustain erosions, whereas excessive or spatially dysregulated activity may drive pathological bone formation in osteoarthritis, spondyloarthritis and heterotopic ossification [19–26]. The balance and context of the response therefore matter more than its direction.

Rosavin and salidroside are bioactive constituents associated with *Rhodiola rosea*. Salidroside has been reported to stimulate osteoblast differentiation through BMP-related signaling and to promote osteogenic differentiation via Wnt/β-catenin-associated mechanisms in mesenchymal models [27–30], and rosavin to suppress osteoclastogenesis through NF-κB and MAPK inhibition [31]; both have been reviewed as modulators of bone metabolism [30, 32].

**Fig. 1:**
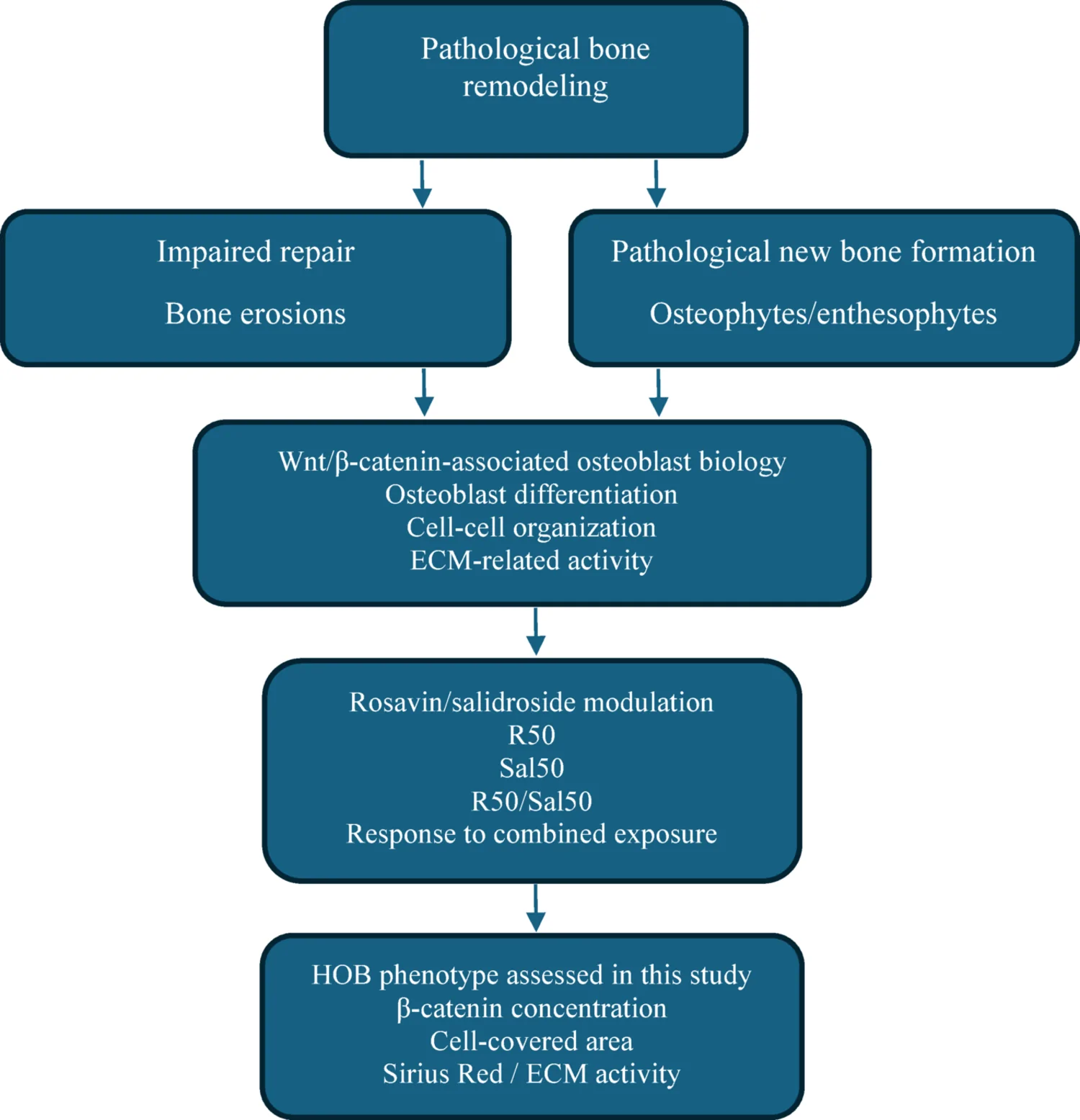
Conceptual framework of rosavin/salidroside-associated modulation of β-catenin-related osteoblast responses in pathological bone remodeling. The figure summarizes the rationale linking dysregulated osteoblast remodeling, Wnt/β-catenin-associated responses and the potential modulatory role of rosavin, salidroside and their combined treatment. Rheumatic diseases represent one clinically important example of pathological bone remodeling, but the present in vitro model should not be interpreted as disease-specific

Experimental work in human osteoblast (HOB) cultures has shown that rosavin modulates BMP-2-associated responses, morphology and cell coverage [33], and that it alters CX3CL1 secretion and morphology-related features under mineralizing conditions [34]. The conceptual framework linking rosavin and salidroside, β-catenin-associated osteoblast organization and pathological bone remodeling is summarized in Fig. 1.

Rosavin and salidroside occur together in *Rhodiola rosea* preparations, and their combined action on osteoblasts has not been examined. Because constituents of plant-derived preparations need not act cumulatively, a combination may not reproduce the effect of either compound alone, and in the context of pathological remodeling the relevant therapeutic objective may be balanced rather than maximal osteoblast activity.

We therefore tested the hypothesis that rosavin, salidroside and their equimolar combination differ in their effect on total β-catenin concentration in HOB cultures under mineralizing conditions, and specifically that the combination does not reproduce the effect of either single compound. The primary endpoint was total β-catenin concentration at days 7, 14 and 21; image-based cell-covered area and collagen-related matrix signal were secondary and supportive endpoints, respectively. Pathological bone remodeling provides the rationale for the question; the model itself is a non-diseased osteoblast monoculture.

## Materials and methods

### Ethics statement and cell source

This study did not involve human participants; no biological material or personal data were collected, received or processed by the authors. The model consisted of commercially purchased primary human osteoblasts (HOBs; PromoCell, Heidelberg, Germany; Cat. No. C-12720), isolated by the manufacturer from trabecular bone of the femur adjacent to the knee or hip joint. According to the supplier’s documentation the donor material was obtained with written informed consent under an ethics-approved protocol, and each lot is released only after testing negative for HIV-1, HIV-2, hepatitis B and C and for bacterial, fungal and mycoplasma contamination. The authors received anonymized lot documentation only.

Under Polish law, research performed exclusively on commercially obtained, permanently anonymized human cell preparations does not constitute a medical experiment and is not subject to bioethics committee opinion; ethics approval was therefore not required and none was sought. The research was nonetheless conducted in accordance with the ethical standards of the 1964 Declaration of Helsinki and its later amendments, as revised by the 75th General Assembly of the World Medical Association (Helsinki, October 2024).

No separate authentication was performed by the authors, as the material is a primary, non-immortalized human cell preparation rather than a continuous cell line; osteoblastic identity and function were verified operationally by the response to mineralization medium and by collagen-related matrix staining.

### Human osteoblast culture and experimental design

Cells were expanded in 25 cm² flasks in Osteoblast Growth Medium (Basal Medium, PromoCell C-27001; SupplementMix, C-39615) with 1% penicillin–streptomycin (Sigma-Aldrich, St. Louis, MO, USA; P0781), at 37 °C in a humidified atmosphere with 5% CO₂ and medium renewed every three days; detachment used DetachKit (PromoCell C-41200). Passage-7 cells were seeded into 12-well plates at 84,000 cells per well (approximately 22,000 cells/cm²).

Four independently cultured and treated wells were analyzed for each experimental condition and time point in the β-catenin ELISA. Each well was seeded, cultured, treated, harvested and lysed separately and constituted the well-level experimental replicate; the well was also the unit to which treatment was allocated. The observations were not repeated readings of a single lysate.

All wells originated from the same commercial primary HOB preparation and the same experimental series. The study therefore did not assess donor-to-donor or between-experiment variability, and the analyses below apply to the effect of treatment on wells within this series rather than to generalization across donors or independent culture runs. Confirmatory interpretation requires replication using separate donor lots in independently conducted experiments. The complete well-level dataset is provided in Online Resource 1 (Table S1).

### Experimental groups and treatment protocol

After initial culture, the medium was changed according to experimental allocation. Five conditions were used, as summarized in Table 1: a growth medium control (GM), a mineralization medium control (MM), and mineralization medium supplemented with rosavin 50 µM (R50), salidroside 50 µM (Sal50) or both compounds together (R50/Sal50).

GM was maintained in Osteoblast Growth Medium as above; MM in Osteoblast Mineralization Medium (PromoCell C-27020) with its dedicated SupplementMix (C-39616) and no test compound. R50, Sal50 and R50/Sal50 received mineralization medium supplemented with the relevant compound or combination. Medium was changed every three days [35–37].

**Table 1:** Experimental groups and readouts

Group	Culture condition	Treatment
GM	Growth medium	None
MM	Mineralization medium	None
R50	Mineralization medium	Rosavin 50 µM
Sal50	Mineralization medium	Salidroside 50 µM
R50/Sal50	Mineralization medium	Rosavin 50 µM + salidroside 50 µM

All five conditions were run in parallel within a single experimental series. Day 0 was sampled before rosavin or salidroside was added and therefore represents a pre-treatment baseline for every condition; at that point the four conditions maintained in mineralization medium were still identical. Total β-catenin (four independently cultured, treated, harvested and lysed wells per condition per time point) and image-based cell-covered area were assessed at days 7, 14 and 21. Sirius Red staining was performed at days 0, 7, 14 and 21, in one well per condition at days 0 and 7 and in two wells at days 14 and 21. GM, growth medium control; MM, mineralization medium control; R50, rosavin 50 µM; Sal50, salidroside 50 µM; R50/Sal50, combined rosavin 50 µM and salidroside 50 µM.

### Preparation of rosavin solution

Rosavin (Sigma-Aldrich, St. Louis, MO, USA; SML0336) was dissolved in sterile phosphate-buffered saline (PBS; ThermoFisher Scientific, San Diego, CA, USA; 14190169) to a 10 mM stock and diluted freshly in mineralization medium immediately before use, to a final 50 µM in R50 and R50/Sal50. The stock thus contributed a vehicle load of 0.5% (v/v) PBS to those two conditions only; salidroside was dissolved directly in medium and contributed none. Because 0.5% isotonic PBS is a negligible dilution of medium components, no separate vehicle control was included.

### Preparation of salidroside solution

Salidroside was an analytical standard (Sigma-Aldrich/Supelco, Merck KGaA, Darmstadt, Germany; 43866; CAS 10338-51-9; molecular weight 300.30 g/mol; purity ≥ 98.0% by HPLC per the manufacturer). It was freshly dissolved in mineralization medium immediately before supplementation to a final 50 µM in Sal50 and R50/Sal50; in the combined condition both compounds were added to the same medium at 50 µM each.

### Time points and sample collection

Day 0 was the baseline time point, sampled before rosavin or salidroside was added; the compounds were introduced after 24 h of culture under mineralizing conditions. Sirius Red staining was performed at days 0, 7, 14 and 21, whereas total β-catenin and image-based cell-covered area were assessed at days 7, 14 and 21. Phase-contrast images were also acquired at day 0 as pre-treatment baseline material; these are not shown in Fig. 3, which presents days 7 to 21.

### β-catenin ELISA

Total β-catenin was quantified in cell lysates with a Human β-Catenin ELISA kit (Abcam, Waltham, MA, USA; ab275100) per the manufacturer’s protocol, absorbance read at 450 nm (ASYS UVM 340, ASYS Hitech GmbH, Eugendorf, Austria). Concentrations were interpolated from a standard curve fitted to the kit standards and expressed in ng/mL. Lysates were assayed undiluted and not normalized to total protein or DNA content. Per-well absorbances and interpolated concentrations are given in Online Resource 1 (Table S1).

### Phase-contrast microscopy and image-based analysis

Images were acquired on an inverted phase-contrast microscope (Zeiss Primovert, Jena, Germany) with a Zeiss Axiocam ERc 5 s camera, under identical settings for all conditions and time points. Cell-covered area was estimated semi-quantitatively by an area-fraction workflow [38, 39] in Fiji/ImageJ version 1.54f (National Institutes of Health, Bethesda, MD, USA).

A duplicate-file check confirmed that no image entered the analysis twice. A fixed central region of interest of constant dimensions was applied to every image, excluding the band containing the scale bar. Images were converted to 8-bit grayscale, downscaled, locally contrast-enhanced, background-corrected by rolling-ball subtraction and binarized with a single global threshold derived from the pooled intensity histogram of the whole image set, so that identical parameters were applied throughout and no threshold was adjusted for individual images. The readout was area fraction, the percentage of the region occupied by cell-associated structures, interpreted as a proxy for cell-covered area rather than a cell count.

Image acquisition and analysis were not blinded to condition, as the files retained their group labels; the readout was nonetheless produced by an automated workflow applying identical parameters to every image, with no operator-adjusted thresholds or manual region selection. Panels in Fig. 3 are shown in grayscale, with any brightness or contrast adjustment applied identically to all panels and not used for quantification.

### Sirius Red staining

Collagen-related extracellular matrix (ECM) activity was assessed by Sirius Red staining [40]. Cultures were washed with calcium–magnesium phosphate-buffered saline (ThermoFisher Scientific; 14040174), incubated with 0.1% Sirius Red (Direct Red 80; Sigma-Aldrich; 365548) for 1 h at 37 °C, washed three times with 10 mM HCl, and bound dye dissolved in 0.1 M NaOH; absorbance was read at 540 nm with blank correction against NaOH controls.

Results were analyzed as blank-corrected optical density (OD). Staining was performed in one well per condition at days 0 and 7 and in two wells per condition at days 14 and 21. Because blank-corrected absorbance already differed between conditions at day 0, before any compound had been added, and because the two readings obtained at days 14 and 21 differed by a consistent offset in the same direction across all five conditions, this endpoint was reported per individual well, used only to confirm the presence of collagen-related matrix activity, and excluded from between-group comparison and from statistical testing.

### Statistical analysis

Analyses were performed in GraphPad Prism version 10 (GraphPad Software, Boston, MA, USA; RRID: SCR_002798); image analysis used Fiji/ImageJ version 1.54f (RRID: SCR_003070). Data organization was performed in Microsoft Excel for Microsoft 365 (Microsoft Corporation, Redmond, WA, USA).

Data are summarized as mean ± standard deviation (SD). Group variances were markedly unequal for both quantitative endpoints, the largest-to-smallest ratio reaching 272, 20 and 20 at days 7, 14 and 21, and field numbers were unequal for the image-based endpoint. Welch’s heteroscedastic one-way ANOVA was therefore pre-specified as the primary omnibus test, applied separately at each time point, with Games–Howell post-hoc comparisons controlling the family-wise error rate within each time point; the three omnibus tests were adjusted across time points using the Holm procedure. Effect sizes are reported as η² for omnibus comparisons and as Hedges’ *g* with 95% confidence intervals for pairwise contrasts.

Fisher’s one-way ANOVA, the Kruskal–Wallis test, analyses of log₁₀-transformed concentrations and a two-way ANOVA with treatment and time as fixed factors were pre-specified sensitivity analyses (Online Resource 1, Table S2). Because β-catenin was not normalized to protein content, group-mean β-catenin was correlated with group-mean cell-covered area across all treatment-by-time combinations. Two-sided p values below 0.05 were considered significant; no category of borderline significance was applied, and exact p values with effect sizes are reported throughout. Sirius Red was not tested statistically. All analyses were recomputed from the well-level data and independently verified by a statistician acknowledged below.

### Reporting standards

The EQUATOR Network library was consulted. No fully endorsed EQUATOR reporting guideline currently exists for in vitro cell-culture studies; the applicable entry in the laboratory and in vitro category is the CRIS Statement (Checklist for Reporting In-vitro Studies) [41], which this report follows. Because CRIS does not address analytical transparency in detail, reporting was additionally aligned with the MDAR (Materials, Design, Analysis, Reporting) Framework [42] with respect to reagent identification, replication structure, applicability of blinding and randomization, statistical reporting and data availability.

## Results

### Total β-catenin concentration

**Fig. 2:**
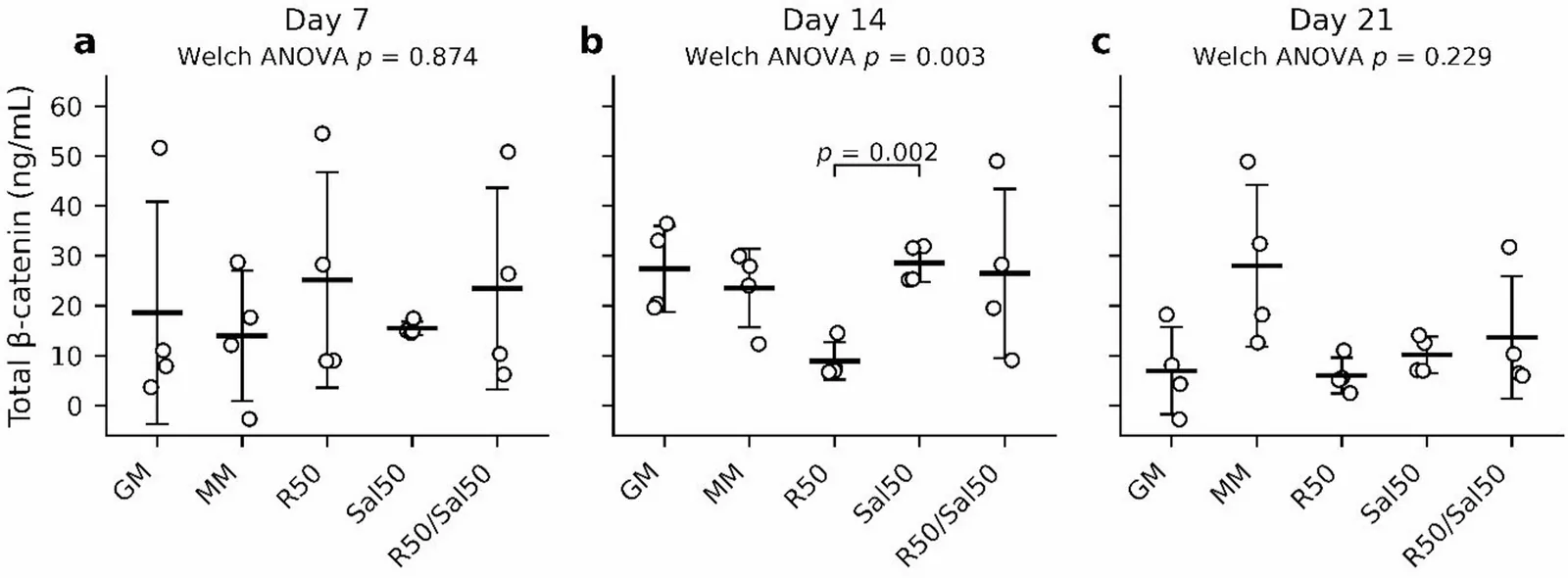
Total β-catenin concentration in HOB cultures at days 7 (**a**), 14 (**b**) and 21 (**c**). Open circles show the individual values of the four independently cultured, treated, harvested and lysed wells analyzed per condition; horizontal bars show condition means and whiskers show SD. The result of the pre-specified primary omnibus test is given above each panel. The bracket in **b** indicates the Games–Howell post-hoc comparison of Sal50 with R50. Abbreviations as in Table 1

Total β-catenin concentrations for all conditions and time points are given in Table 2, individual well values in Fig. 2, and the complete dataset in Online Resource 1 (Table S1).

Concentrations differed between conditions at day 14 (Welch ANOVA F(4, 7.18) = 11.69, *p* = 0.003; Holm-adjusted across the three time points, *p* = 0.009; η² = 0.43). R50 was the lowest condition at 8.9 ± 3.8 ng/mL, 62% below the mineralization control and 2.6- to 3.2-fold below every other condition (Sal50 28.5 ± 3.8; GM 27.4 ± 8.6; R50/Sal50 26.5 ± 16.9; MM 23.5 ± 7.9 ng/mL). In Games–Howell post-hoc testing, Sal50 versus R50 was the only contrast retaining significance after correction (mean difference 19.6 ng/mL, 95% CI 13.1–26.1; *p* = 0.002; Hedges’ *g* = 4.54, 95% CI 1.70–7.38). R50/Sal50 versus R50 lay in the same direction but did not reach significance (*p* = 0.41), the combined condition showing the widest dispersion of any group (SD 16.9 ng/mL).

No difference was detected at day 7 (Welch ANOVA *p* = 0.87) or day 21 (*p* = 0.23), and the ordering of condition means at these time points is not interpreted. Sensitivity analyses (Online Resource 1, Table S2) were concordant with the primary result at days 7 and 14; at day 21 Fisher’s ANOVA gave *p* = 0.048 against a primary result of *p* = 0.23, a discordance consistent with the variance heterogeneity at that time point and a further reason not to interpret the day-21 comparison.

Because β-catenin was measured without normalization to protein content, group-mean concentrations were compared with group-mean cell-covered area across all fifteen treatment-by-time combinations. The two were not positively associated (Pearson *r* = − 0.35, 95% CI − 0.73 to 0.20, *p* = 0.21; Spearman ρ = −0.33, *p* = 0.24), cell-covered area accounting for approximately 12% of the variance in group-mean β-catenin. The measured signal is therefore unlikely to be a direct reflection of the number of cells present.

**Table 2:** Total β-catenin concentration and primary between-condition comparisons

Day	GM	MM	R50	Sal50	R50/Sal50	Welch ANOVA	Holm *p*	η²
7	18.6 ± 22.3	14.0 ± 13.1	25.2 ± 21.6	15.5 ± 1.4	23.4 ± 20.2	F(4, 6.07) = 0.29, *p* = 0.874	0.874	0.08
14	27.4 ± 8.6	23.5 ± 7.9	8.9 ± 3.8	28.5 ± 3.8	26.5 ± 16.9	F(4, 7.18) = 11.69, *p* = 0.003	0.009	0.43
21	7.0 ± 8.8	28.0 ± 16.2	6.0 ± 3.6	10.2 ± 3.7	13.6 ± 12.3	F(4, 7.10) = 1.82, *p* = 0.229	0.459	0.45

Mean ± SD of interpolated β-catenin concentration (ng/mL); *n* = 4 independently cultured, treated, harvested and lysed wells per condition and time point. Primary analysis: Welch’s heteroscedastic one-way ANOVA, Holm-adjusted across the three time points. Games–Howell post-hoc testing identified a single significant contrast, Sal50 versus R50 at day 14 (mean difference 19.6 ng/mL, 95% CI 13.1–26.1; *p* = 0.002). Sensitivity analyses and full post-hoc comparisons are given in Online Resource 1, Table S2. Abbreviations as in Table 1

### Image-based cell-covered area

**Fig. 3:**
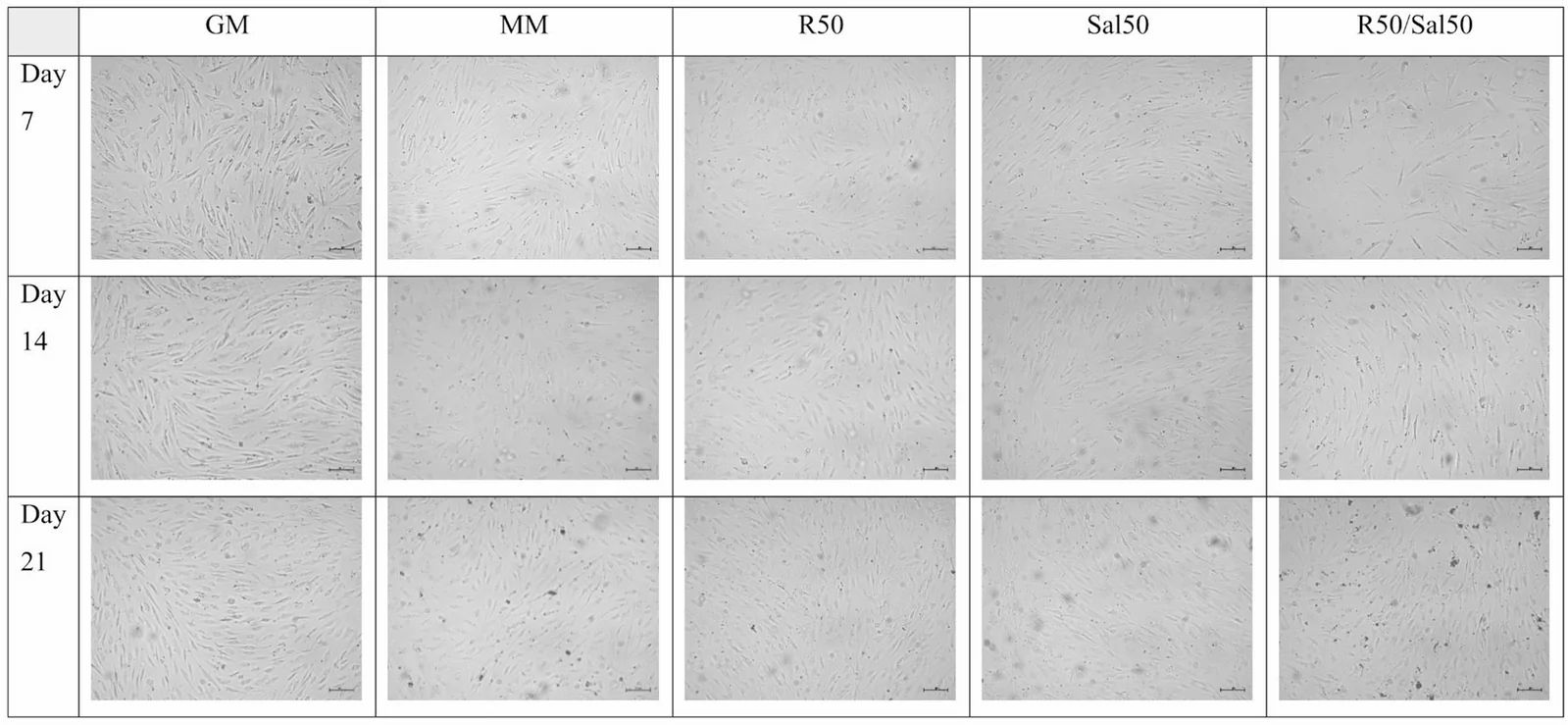
Representative HOB morphology after treatment. Representative phase-contrast microscopy images of HOB cultures at days 7, 14 and 21. Columns show GM, MM, R50, Sal50 and R50/Sal50; rows show consecutive post-treatment time points. Quantitative image-based area-fraction results are presented in Table 3. Scale bar: 50 μm. Representative images are presented in grayscale for visual consistency; quantitative area-fraction analysis was performed using the image-processing workflow described in Sect. 2.8. Abbreviations as in Table 1

A total of 79 unique phase-contrast fields were analyzed, corresponding to a median of five fields per condition and time point; the number of usable fields differed between conditions, and this inequality was accommodated by the use of Welch’s ANOVA. Condition means and the primary omnibus tests are given in Table 3, and representative images in Fig. 3.

Between-condition differences were detected at days 7 and 21. At day 7 the highest area-fraction values were observed in GM, while R50, Sal50 and MM showed intermediate-to-high values and R50/Sal50 was markedly lower. No difference was detected at day 14, and condition means at that time point are not ranked. At day 21 GM again showed the highest area fraction, followed by R50, Sal50 and MM, with R50/Sal50 lowest.

At all three time points R50/Sal50 showed both the lowest mean area fraction and by far the widest dispersion, with SD of 21.5–23.4% points against 0.8–12.2 in every other condition. This indicates heterogeneity of cell coverage across the culture surface under combined treatment rather than a uniform reduction, and is consistent with the representative images in Fig. 3.

**Table 3:** Image-based cell-covered area fraction and primary between-condition comparisons

Day	GM	MM	R50	Sal50	R50/Sal50	Welch *p*	Holm *p*
7	98.1 ± 1.8	87.0 ± 5.4	86.8 ± 3.0	84.5 ± 6.6	54.5 ± 23.4	< 0.001	< 0.001
14	93.0 ± 11.6	69.8 ± 3.4	73.5 ± 12.2	78.2 ± 9.0	70.4 ± 22.3	0.085	0.085
21	99.0 ± 0.8	91.7 ± 4.0	96.1 ± 2.6	94.2 ± 4.4	77.2 ± 21.5	0.020	0.040

Mean ± SD of area fraction (%), used as a semi-quantitative proxy of cell-covered area rather than a cell count; 79 unique fields analyzed in total, with unequal numbers per condition. Primary analysis: Welch’s heteroscedastic one-way ANOVA, Holm-adjusted across the three time points. Fisher’s ANOVA and Kruskal–Wallis results are given in Online Resource 1, Table S2. Abbreviations as in Table 1

### Collagen-related matrix signal

Sirius Red staining confirmed that collagen-related matrix activity was present in all conditions and increased over the culture period, blank-corrected absorbance at day 21 exceeding day-0 values in every condition. Individual well readings are given in Online Resource 1 (Table S3).

Two features of this dataset preclude comparison between conditions. First, at day 0 the four conditions maintained in mineralization medium were still identical, because the compounds were added only after that time point; their blank-corrected absorbance nevertheless ranged from 0.134 to 0.454, a 3.4-fold spread between nominally equivalent wells. With one well stained per condition at day 0, this range is a direct estimate of well-to-well variability in the assay, and it exceeds most of the between-condition differences seen at later time points. Second, the two wells stained at days 14 and 21 differed by a consistent offset in the same direction across all five conditions, of 0.27 and 0.26 OD units respectively, while the corresponding blanks were comparable. This endpoint is therefore reported descriptively and per well, and no between-condition statement is made.

## Discussion

This study compared rosavin, salidroside and their equimolar combination in primary human osteoblast cultures under mineralizing conditions. One result was robust to correction for both multiple pairwise comparisons and multiple time points: at day 14, cultures exposed to rosavin alone contained approximately three times less total β-catenin than any other condition. The combination did not reproduce this reduction, nor did it maximize cell coverage; it showed the lowest and most variable area fraction of any condition at all three time points. The two constituents therefore do not act interchangeably on human osteoblasts, and the effect of the combination cannot be predicted from either compound alone. The present design cannot establish the nature of the interaction, and no claim about synergy or antagonism is made.

β-catenin is a central mediator of canonical Wnt signaling in osteoblast differentiation, survival and matrix-related activity [11, 12, 14, 15], but it should not be read as a linear marker in which higher values indicate a more favorable phenotype: it also participates in cadherin-associated cell–cell adhesion, so its total concentration reflects both signaling and structural pools.

Two considerations bear on how the day-14 reduction should be read. First, the assay quantifies the total cellular pool and cannot separate the transcriptionally active fraction from the cadherin-bound structural fraction, so a change in total concentration may reflect redistribution between compartments rather than altered pathway activity. Second, canonical Wnt activity is not required at a constant level throughout osteoblast maturation: sustained β-catenin signaling supports commitment and proliferation, whereas its attenuation accompanies the transition towards matrix production. A lower total concentration accompanied by preserved cell coverage is therefore not straightforwardly an adverse observation. These interpretations cannot be distinguished without measurement of active and non-phosphorylated β-catenin, nuclear localization and downstream Wnt targets such as *AXIN2*, *LEF1*/*TCF*, *RUNX2*, *ALP* and *OCN*, none of which was undertaken here.

The mineralization control showed the numerically highest concentration at day 21, but no between-condition difference was detected at that time point and this is not interpreted further.

These observations are consistent with earlier reports of rosavin- and salidroside-associated modulation of osteoblast responses [33, 34], although the direction of the effect depends on the endpoint and time point examined. Salidroside has been reported to stimulate osteoblast differentiation through BMP-related signaling [27] and to promote osteogenic differentiation through Wnt/β-catenin-associated mechanisms in adipose-derived stromal cells [28].

The combination, rather than either isolated compound, corresponds to the exposure that occurs with commercial Rhodiola rosea preparations, in which rosavin and salidroside are always present together. This makes the behavior of the combined condition the more clinically pertinent observation. Within the framework set out in Sect. 1, in which balanced rather than maximal osteoblast activity is the relevant objective in pathological remodeling, the finding that combined exposure left total β-catenin within the range of both control conditions while rosavin alone did not defines a question worth testing rather than a demonstrated benefit.

Three considerations restrain any stronger reading. The pairwise comparison between the combination and rosavin alone did not itself reach significance. The combination was associated with the lowest and most variable cell coverage of any condition, which is not an obviously favorable phenotype. And a single equimolar ratio cannot characterize an interaction. What the data support is the narrower statement that the two constituents are not equivalent and that their combination is not predictable from either alone.

Wnt-dependent bone regulation has been examined repeatedly in this journal, consistently at the level of circulating regulators. Serum Dickkopf-1, sclerostin and transforming growth factor-β1 were lower in spondyloarthritis than in healthy controls, Dickkopf-1 correlating positively with C-reactive protein and negatively with chronic sacroiliac changes, and sclerostin negatively with active inflammatory lesions [43]. Sclerostin likewise correlated negatively with inflammation and radiographic spinal damage in axial spondyloarthritis [44], and Dickkopf-1 was lower in ankylosing spondylitis and unaffected by anti-tumour necrosis factor therapy [45]. These reports show that Wnt antagonist levels track disease activity, but they measure upstream regulators in serum and cannot show what happens to β-catenin inside the bone-forming cell.

The present report adds three elements to that literature. It measures total β-catenin directly in primary human osteoblasts rather than inferring pathway status from circulating antagonists. It tests two constituents of a widely available plant preparation, and their combination, rather than a single agent. And it makes the complete primary dataset available so that the analysis can be reproduced.

The disease-related relevance should nonetheless be framed cautiously. An HOB monoculture reproduces no specific rheumatic disease entity, although it addresses osteoblast and Wnt/β-catenin processes central to pathological remodeling, in which both insufficient repair and excessive or ectopically directed bone formation occur [7–10, 19–26]. These data do not demonstrate efficacy in rheumatic disease and are not disease-specific; Wnt-associated remodeling is also relevant to metabolic and cancer-associated contexts [46–48].

The effects of the two compounds are not explained by total β-catenin alone: R50 and Sal50 maintained high cell coverage, whereas R50/Sal50 was distinctly more variable despite a comparable β-catenin concentration. BMP/Smad signaling is the most immediate additional candidate, given the reported rosavin-associated BMP-2 modulation in HOB cultures and the BMP-dependence of salidroside-induced differentiation [27, 33]. Further candidates are identified in Sect. 4.2 as measurements to be made rather than mechanisms inferred here.

### Limitations

The study was conducted as a single culture series using one commercial HOB preparation. Within that series each condition was allocated to four independently cultured, treated, harvested and lysed wells, which constitute the experimental units and support the analyses reported; what the design cannot estimate is donor-to-donor and between-series variance, and the findings therefore require replication in independent series using separate donor lots. Post hoc, the design provided approximately 77% power for the day-14 omnibus comparison at the observed η² of 0.43, and the smallest difference detectable between two conditions was 6.5 ng/mL.

β-catenin was measured as total protein in unnormalized lysates, without the active, non-phosphorylated or nuclear fractions or downstream targets, so no statement about pathway activity is possible. Area fraction is a semi-quantitative proxy sensitive to morphology and spreading, and acquisition and analysis were not blinded. Sirius Red comprised one to two wells per condition and is descriptive only. A single concentration and a single equimolar ratio were tested, no viability assay was performed, and no vehicle control was included. Finally, the model is a monoculture with no immune, synovial or osteoclastic compartment; it reproduces no rheumatic disease and is not evidence of efficacy.

### Future perspectives

Future work should test whether this profile is reproduced in independent biological series and under disease-relevant conditions: HOB cultures exposed to TNF-α, IL-1β or IL-6, oxidative stress models, and co-cultures with osteoclast precursors, synoviocytes or chondrocytes. Mechanistic validation should include active and nuclear β-catenin and Wnt target genes, BMP/Smad, PI3K/Akt and ERK/MAPK signaling, Nrf2/HO-1, NF-κB and IL-6, and the OPG/RANKL balance. Concentration–response designs with parallel viability assessment are required before any interaction can be characterized.

### Clinical and ethical implications

*Rhodiola rosea* extracts standardized to rosavin and salidroside are sold without prescription and used by patients with rheumatoid arthritis, spondyloarthritis and osteoarthritis, often without the treating rheumatologist’s knowledge. Preclinical findings in this area reach the public quickly and are readily read as therapeutic endorsement. These results provide no basis for clinical use, and the direction of the observed effect on β-catenin supports no prediction about bone in patients. The one observation with a practical bearing is cautionary: the two constituents did not behave equivalently, so the assumption that a standardized extract can be characterized by its individual marker compounds, or that more of any one is preferable, is not supported.

Ethically, the study used commercially obtained, permanently anonymized human-derived cells for which donor consent was documented by the supplier, and the authors had no access to identifying information. The in vitro design also avoided the use of experimental animals at this exploratory stage, consistent with the principle of replacement; the animal and co-culture models identified above are appropriate only once the present observations have been shown to be reproducible.

## Conclusions

In primary human osteoblast cultures under mineralizing conditions, rosavin alone was associated with a total β-catenin concentration approximately three-fold lower than that of salidroside and of both control media at day 14, a difference that survived correction for multiple comparisons and multiple time points. Combining rosavin with salidroside did not reproduce this reduction, and the combination was also associated with the lowest and most variable cell coverage of any condition. The two *Rhodiola rosea* constituents therefore do not act interchangeably on human osteoblasts.

These observations derive from a single culture series and concern total rather than active β-catenin. They support no mechanistic or clinical conclusion, but they identify a specific question — whether the constituents of standardized *Rhodiola* preparations act independently on bone-forming cells — that warrants replication in independent biological series and testing under inflammatory and disease-relevant conditions.

## Disclaimer

No part of this article has been copied from any other source or published elsewhere, in whole or in part. The study concept, experimental design, laboratory work, data analysis, interpretation and writing were carried out entirely by the authors. AI-based tools were used only for language and copy-editing assistance in author-written text; no part of the content was generated by artificial intelligence. All content was reviewed, verified and approved by the authors, who take full responsibility for the entire manuscript.

## Supplementary Information

Below is the link to the electronic supplementary material.

Supplementary Material 1

## Data Availability

All data supporting the findings of this study are provided within the article and its Online Resource. The complete well-level β-catenin dataset, comprising raw absorbance values and interpolated concentrations for every well, the individual Sirius Red readings and the full set of sensitivity analyses, is available in Online Resource 1. No other datasets were generated or analyzed during this study.
