# Supplementary material for: Rosavin, but not its combination with salidroside, lowers total β-catenin in human osteoblast cultures: an exploratory in vitro study under mineralizing conditions
Source: Rheumatol Int. 2026 Aug 17;46(9):239. doi: 10.1007/s00296-026-06278-y (PMC13481743; doi:10.1007/s00296-026-06278-y)
Supplement: Supplementary file 1 [file 296_2026_6278_MOESM1_ESM.docx]

**Online Resource 1**

*Supplementary data and statistical analyses for: Rosavin, but not its combination with salidroside, lowers total β-catenin in human osteoblast cultures: an exploratory in vitro study under mineralizing conditions*

This file contains the complete primary dataset for the quantitative endpoints and the pre-specified sensitivity analyses referred to in the main text. Every statistic reported in the article can be recomputed from Table S1 (total β-catenin) and Table S3 (Sirius Red). Abbreviations: GM, growth medium control; MM, mineralization medium control; R50, rosavin 50 µM; Sal50, salidroside 50 µM; R50/Sal50, combined rosavin 50 µM and salidroside 50 µM.

**Table S1 Complete well-level dataset for total β-catenin**

**(a) Raw absorbance at 450 nm**

| **Day** | **Well** | **GM** | **MM** | **R50** | **Sal50** | **R50/Sal50** |
| --- | --- | --- | --- | --- | --- | --- |
| 7 | 1 | 0.692 | 0.443 | 0.229 | 0.294 | 0.418 |
| 7 | 2 | 0.217 | 0.323 | 0.228 | 0.289 | 0.199 |
| 7 | 3 | 0.251 | 0.263 | 0.438 | 0.293 | 0.243 |
| 7 | 4 | 0.171 | 0.102 | 0.723 | 0.321 | 0.683 |
| 14 | 1 | 0.490 | 0.456 | 0.289 | 0.405 | 0.438 |
| 14 | 2 | 0.353 | 0.434 | 0.213 | 0.406 | 0.343 |
| 14 | 3 | 0.527 | 0.392 | 0.207 | 0.478 | 0.230 |
| 14 | 4 | 0.344 | 0.265 | 0.204 | 0.474 | 0.663 |
| 21 | 1 | 0.329 | 0.483 | 0.191 | 0.208 | 0.201 |
| 21 | 2 | 0.178 | 0.329 | 0.158 | 0.267 | 0.476 |
| 21 | 3 | 0.219 | 0.662 | 0.187 | 0.207 | 0.196 |
| 21 | 4 | 0.101 | 0.268 | 0.251 | 0.284 | 0.243 |

**(b) Interpolated β-catenin concentration (ng/mL)**

| **Day** | **Well** | **GM** | **MM** | **R50** | **Sal50** | **R50/Sal50** |
| --- | --- | --- | --- | --- | --- | --- |
| 7 | 1 | 51.66 | 28.72 | 9.00 | 14.99 | 26.42 |
| 7 | 2 | 7.90 | 17.66 | 8.91 | 14.53 | 6.24 |
| 7 | 3 | 11.03 | 12.14 | 28.26 | 14.90 | 10.29 |
| 7 | 4 | 3.66 | -2.69 | 54.51 | 17.48 | 50.83 |
| 14 | 1 | 33.05 | 29.92 | 14.53 | 25.22 | 28.26 |
| 14 | 2 | 20.43 | 27.89 | 7.53 | 25.31 | 19.51 |
| 14 | 3 | 36.46 | 24.02 | 6.98 | 31.94 | 9.10 |
| 14 | 4 | 19.60 | 12.32 | 6.70 | 31.57 | 48.98 |
| 21 | 1 | 18.22 | 32.40 | 5.50 | 7.07 | 6.43 |
| 21 | 2 | 4.31 | 18.22 | 2.46 | 12.51 | 31.76 |
| 21 | 3 | 8.08 | 48.89 | 5.14 | 6.98 | 5.96 |
| 21 | 4 | -2.79 | 12.60 | 11.03 | 14.07 | 10.29 |

Each observation originated from a separately seeded, cultured, treated, harvested and lysed well; the well was the experimental unit and also the unit to which treatment was allocated. All wells derived from the same commercial primary human osteoblast preparation and the same experimental series. Concentrations in panel (b) were interpolated from the standard curve fitted to the kit standards; all statistics reported in the main text and in Table S2 were computed from these values. Two of the 60 wells (day 7, well 4, MM; day 21, well 4, GM) had absorbance below the lowest calibrator and therefore yielded interpolated values below zero; these were retained unchanged rather than censored, so that group means are not biased upward. No observation was modified or excluded.

**Table S2 Pre-specified sensitivity analyses and supplementary statistics**

**(a) Total β-catenin: between-condition comparisons at each time point**

| **Analysis** | **Day 7** | **Day 14** | **Day 21** |
| --- | --- | --- | --- |
| Welch ANOVA (primary) | F(4, 6.07) = 0.29, p = 0.874 | F(4, 7.18) = 11.69, p = 0.003 | F(4, 7.10) = 1.81, p = 0.229 |
| Holm-adjusted p (three time points) | 0.874 | 0.009 | 0.459 |
| Fisher one-way ANOVA | F(4, 15) = 0.31, p = 0.867 | F(4, 15) = 2.88, p = 0.059 | F(4, 15) = 3.09, p = 0.048 |
| Kruskal–Wallis | H(4) = 0.89, p = 0.927 | H(4) = 8.93, p = 0.063 | H(4) = 9.00, p = 0.061 |
| Fisher ANOVA, log₁₀ scale | not applicable | F(4, 15) = 5.24, p = 0.008 | not applicable |
| Welch ANOVA, log₁₀ scale | not applicable | F(4, 6.84) = 7.63, p = 0.011 | not applicable |

Welch’s heteroscedastic one-way ANOVA was pre-specified as the primary omnibus test because group variances were markedly unequal (largest-to-smallest variance ratio 272, 20 and 20 at days 7, 14 and 21). The remaining analyses are pre-specified sensitivity analyses. Logarithmic transformation was not applicable at days 7 and 21 because one well at each of these time points had an interpolated value below zero (Table S1). At day 21 Fisher’s ANOVA is discordant with the primary analysis, consistent with the variance heterogeneity at that time point; the day-21 comparison is therefore not interpreted in the main text.

**(b) Total β-catenin, day 14: Games–Howell post-hoc comparisons**

| **Contrast** | **Mean difference (ng/mL)** | **95% CI** | **t** | **df** | **Adjusted p** |
| --- | --- | --- | --- | --- | --- |
| R50 vs Sal50 | -19.58 | -26.06 to -13.09 | 7.38 | 6.00 | 0.002 |
| GM vs R50 | 18.45 | 5.50 to 31.39 | 3.92 | 4.09 | 0.072 |
| MM vs R50 | 14.60 | 2.82 to 26.38 | 3.35 | 4.29 | 0.111 |
| R50 vs R50/Sal50 | -17.53 | -43.79 to 8.74 | 2.02 | 3.29 | 0.412 |
| MM vs Sal50 | -4.97 | -16.75 to 6.80 | 1.14 | 4.30 | 0.782 |
| GM vs MM | 3.85 | -10.47 to 18.16 | 0.66 | 5.95 | 0.959 |
| MM vs R50/Sal50 | -2.92 | -28.29 to 22.44 | 0.31 | 4.24 | 0.997 |
| Sal50 vs R50/Sal50 | 2.05 | -24.21 to 28.31 | 0.24 | 3.29 | 0.999 |
| GM vs Sal50 | -1.13 | -14.07 to 11.82 | 0.24 | 4.10 | 0.999 |
| GM vs R50/Sal50 | 0.92 | -24.43 to 26.27 | 0.10 | 4.46 | 1.000 |

Games–Howell testing controls the family-wise error rate within the time point and does not assume equal variances. Only Sal50 versus R50 retained significance after correction (Hedges’ g = 4.54, 95% CI 1.70–7.38). No comparison reached significance at day 7 or day 21.

**(c) Total β-catenin: two-way ANOVA with treatment and time as fixed factors**

| **Source** | **Sum of squares** | **df** | **F** | **p** | **Partial η²** |
| --- | --- | --- | --- | --- | --- |
| Treatment | 545.1 | 4 | 0.81 | 0.523 | 0.067 |
| Time | 1026.5 | 2 | 3.07 | 0.056 | 0.120 |
| Treatment × time | 2152.4 | 8 | 1.61 | 0.150 | 0.222 |
| Residual | 7531.5 | 45 |  |  |  |

Balanced design, n = 4 wells per treatment × time cell. The treatment × time interaction corresponds to a large effect size (partial η² = 0.222; Cohen’s f = 0.53) but did not reach statistical significance; post hoc, the design provided approximately 79% power for an interaction of this magnitude. For the day-14 omnibus comparison the design provided approximately 77% power at the observed η² of 0.43, and the smallest difference detectable between two conditions with four wells each was 6.5 ng/mL.

**(d) Image-based cell-covered area: between-condition comparisons at each time point**

| **Day** | **Welch ANOVA (primary)** | **Holm-adjusted p** | **Fisher one-way ANOVA** | **Kruskal–Wallis** |
| --- | --- | --- | --- | --- |
| 7 | p < 0.001 | < 0.001 | p < 0.0001 | p < 0.001 |
| 14 | p = 0.085 | 0.085 | p = 0.203 | p = 0.144 |
| 21 | p = 0.020 | 0.040 | p = 0.028 | p = 0.007 |

Computed from the per-field area-fraction values; 79 unique non-overlapping fields were analysed in total, with unequal numbers of fields per condition, which is why Welch’s ANOVA was pre-specified as the primary test for this endpoint as well. Between-condition differences at days 7 and 21 remain significant after Holm adjustment across the three time points; no difference was detected at day 14.

**Table S3 Individual Sirius Red absorbance readings**

| **Day** | **Replicate** | **NaOH blank** | **GM** | **MM** | **R50** | **Sal50** | **R50/Sal50** |
| --- | --- | --- | --- | --- | --- | --- | --- |
| 0 | 1 | 0.040 | 0.097 | 0.207 | 0.454 | 0.220 | 0.134 |
| 7 | 1 | 0.033 | 0.088 | 0.208 | 0.499 | 0.226 | 0.132 |
| 14 | 1 | 0.040 | 0.102 | 0.218 | 0.462 | 0.218 | 0.142 |
| 14 | 2 | 0.032 | 0.499 | 0.531 | 0.539 | 0.448 | 0.497 |
| 21 | 1 | 0.039 | 0.505 | 0.488 | 0.516 | 0.447 | 0.474 |
| 21 | 2 | 0.040 | 0.559 | 0.540 | 1.032 | 0.858 | 0.726 |

Values under the condition headings are blank-corrected optical density at 540 nm, obtained by subtracting the corresponding NaOH control from the raw absorbance. One well per condition was stained at days 0 and 7 and two wells per condition at days 14 and 21. Two features of this dataset preclude comparison between conditions and are the reason it is reported descriptively and per well in the main text. First, at day 0 the four conditions maintained in mineralization medium (MM, R50, Sal50 and R50/Sal50) were still identical to one another, because the compounds were added only after this time point; their blank-corrected absorbance nevertheless ranged from 0.134 to 0.454, a 3.4-fold spread between nominally equivalent wells, which provides a direct estimate of well-to-well variability in the assay. Second, the two readings obtained at days 14 and 21 differ by a consistent offset in the same direction across all five conditions, of 0.27 and 0.26 optical density units respectively, while the corresponding blanks are comparable; the first day-14 reading is also nearly identical to the day-0 reading in every condition, the largest difference across the five being 0.011 optical density units. This pattern is more compatible with an offset between measurement occasions than with biological variation. No statistical testing was applied to this endpoint.
